# Arrhythmogenic Cardiomyopathy: Towards Genotype Based Diagnoses and Management

**DOI:** 10.1111/jce.16519

**Published:** 2024-12-02

**Authors:** Steven A. Muller, Giorgia Bertoli, Jianan Wang, Alessio Gasperetti, Moniek G. P. J. Cox, Hugh Calkins, Anneline S. J. M. te Riele, Daniel P. Judge, Mario Delmar, Richard N. W. Hauer, Gerard J. J. Boink, Marina Cerrone, J. Peter van Tintelen, Cynthia A. James

**Affiliations:** ^1^ Division of Cardiology, Department of Medicine Johns Hopkins University Baltimore Maryland USA; ^2^ Department of Cardiology University Medical Center Utrecht Utrecht The Netherlands; ^3^ Member of the European Reference Network for Rare low prevalence and complex diseases of the heart: ERN GUARD‐Heart' Amsterdam The Netherlands; ^4^ Leon H. Charney Division of Cardiology New York University Grossman School of Medicine New York New York USA; ^5^ Department of Medical Biology, Amsterdam Cardiovascular Sciences, Amsterdam University Medical Centers University of Amsterdam Amsterdam The Netherlands; ^6^ Department of Cardiology University Medical Centre Groningen Groningen The Netherlands; ^7^ Medical University of South Carolina Charleston South Carolina USA; ^8^ Department of Cardiology, Amsterdam Cardiovascular Sciences, Amsterdam University Medical Centers University of Amsterdam Amsterdam The Netherlands; ^9^ Department of Genetics University Medical Center Utrecht Utrecht The Netherlands

**Keywords:** ACM, ARVC, cardiac arrest/sudden death, clinical:electrophysiology, gene‐specific, genotype‐specific

## Abstract

Arrhythmogenic cardiomyopathy (ACM) is a genetically heterogeneous inherited cardiomyopathy with an estimated prevalence of 1:5000–10 000 that predisposes patients to life‐threatening ventricular arrhythmias (VA) and sudden cardiac death (SCD). ACM diagnostic criteria and risk prediction models, particularly for arrhythmogenic right ventricular cardiomyopathy (ARVC), the most common form of ACM, are typically genotype‐agnostic, but numerous studies have established clinically meaningful genotype‐phenotype associations. Early signs of ACM onset differ by genotype indicating the need for genotype‐specific diagnostic criteria and family screening paradigms. Likewise, risk factors for SCD vary by genetic subtype, indicating that genotype‐specific guidelines for management are also warranted. Of particular importance, genotype‐specific therapeutic approaches are being developed. Results from a randomized controlled trial for flecainide use in ARVC patients are currently pending. Research in a plakophilin‐2‐deficient mouse model suggests this antiarrhythmic drug may be particularly useful for patients with likely pathogenic or pathogenic (LP/P) *PKP2* variants. Additionally, the first gene therapy clinical trials in ARVC patients harboring LP/P *PKP2* variants are currently underway. This review aims to provide clinicians caring for ACM patients with an up‐to‐date overview of the current literature in genotype‐specific natural history of disease and management of ACM patients and describe scientific advances that have led to upcoming clinical trials.

## Graphical abstract

To improve arrhythmogenic cardiomyopathy (ACM) patient care four pillars of ACM research are necessary. The first pillar to improve ACM is cohort studies in which the genotype‐specific natural history of disease is described, and subsequently genotype‐specific risk calculators can be developed, such as the *DSP* and *PLN* risk calculators. The second pillar is the basic and translational studies in which cellular pathways are described and potential genotype‐specific drug targets to slow progression or lower the risk of VA identified, such as flecainide in *PKP2*‐associated ARVC patients. Third, preclinical gene studies can improve ACM patient care to determine which vector has the optimal effect in which specific gene for gene therapy. The efficacy of gene therapy can subsequently be tested to hopefully improve or even cure ACM in clinical trials.

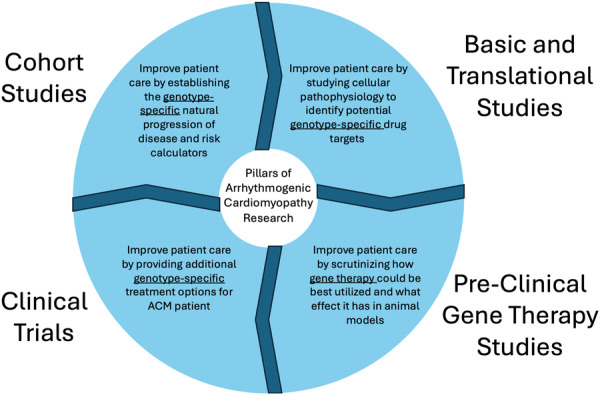

## 1 Introduction

Arrhythmogenic cardiomyopathy (ACM) is a progressive, inherited, cardiomyopathy that predisposes patients to potentially life‐threatening ventricular arrhythmias (VA), heart failure, and sudden cardiac death (SCD) [1]. ACM is most often associated with likely pathogenic or pathogenic (LP/P) variants in genes encoding for desmosomal proteins. Due to the high prevalence of LP/P variants in desmosomal genes among patients with the right dominant subtype of ACM, arrhythmogenic right ventricular cardiomyopathy (ARVC), ACM has sometimes been conceived of as a “disease of the desmosome.” However, LP/P variants in (non‐desmosomal) Transmembrane protein 43 [2] (*TMEM43*), Phospholamban [3] (*PLN*), and Desmin [4] (*DES*) are also causative of ARVC, and variants in both desmosomal and non‐desmosomal genes contribute to left‐dominant (i.e., arrhythmogenic left ventricular cardiomyopathy [ALVC]), right‐dominant ([ARVC]), or biventricular ACM [5].

Following characterization of the genetic architecture of ACM, numerous studies began to explore genotype‐phenotype relationships [6]. Today it is increasingly recognized that there are genotype‐specific differences in phenotypic expression which have important implications for diagnosis, family screening, risk stratification for SCD, and therapeutic management [7, 8, 9]. It is important to note that genotype‐informed management may include consideration of not only the gene in which a LP/P variant is located but sometimes also the specific variant itself [2, 3].

To provide clinicians with an overview of the current literature and highlight the relevance of genotype‐specific diagnosis and management recommendations, we aim to summarize the: (1) genetic architecture and penetrance of ACM; (2) diagnosis of ACM and challenges associated with lack of gene‐specific criteria; (3) management of individuals genetically at‐risk for ACM; (4) gene‐specific approaches to management of patients diagnosed with ACM; and lastly (5) the therapeutic potential of gene therapy (Graphical Abstract). This selection of topics was based on two joint sessions of the European Cardiac Arrhythmia Society (ECAS) and the Heart Rhythm Society, during the 17th Annual ECAS Scientific Congress in Paris, 2024. The sessions were entitled “Arrhythmogenic Cardiomyopathy (ACM): Towards new initially genotype‐based diagnosis I and II.” presented by the authors of this manuscript and chaired by Richard Hauer, J. Peter van Tintelen, and Mario Delmar.

## 2 Genetic Architecture and Penetrance of ACM

Although geographical differences are present, the overall prevalence of ACM is estimated between 1:5000 and 1:10 000 [1] in whom approximately 50% harbor a LP/P variant causal for the disease [10]. ACM inheritance is generally autosomal dominant with age‐related reduced penetrance. Male sex [11], exercise [12], and having multiple LP/P variants [6] have each been shown to contribute to a higher risk of penetrant disease. In the context of family screening, the penetrance in at‐risk relatives (i.e., those who carry the familial variant or are first degree‐relatives of a gene elusive proband) at first evaluation is approximately 33%. In addition to the first evaluation, the yield of serial evaluation at 4 years of follow‐up is approximately 33% [13]. Abnormalities on ECG and/or Holter monitoring (i.e., “electrical” abnormalities) usually precede abnormalities on echocardiography and/or cardiac magnetic resonance imaging (CMR) (i.e., “structural abnormalities”) [13].

Despite being considered a generally autosomal dominant disease, ACM is characterized by incomplete penetrance (i.e., not everyone with the genetic variant will develop ACM) and disease variability (i.e., even within those who develop ACM, there is variability in severity). Of particular importance to this paper, genotype has a vital impact on the clinical course of ACM, and penetrance and the extent of variability in disease expression varies by genotype [6]. The clinical characteristics associated with different ACM subtypes are summarized in Table 1) [14].

**Table 1:** List of ACM genes and associated phenotypes.

ACM gene	Mode of inheritance	Associated phenotype
*PKP2*	Autosomal dominant	Causing up to 80% of classical ARVC cases.
		Male sex and exercise associated with penetrant disease.
*DSP*	Autosomal dominant, some autosomal recessive (associated with Carvajal Syndrome)	Frequent left ventricular involvement. Female sex associated with penetrant disease. “ring”‐like LGE. Acute episodes of myocardial injury
*DSG2*	Autosomal dominant	Data suggest classical ARVC, age of onset maybe early as compared to *PKP2*
*DSC2*	Autosomal dominant, some autosomal recessive	Data suggest classical ARVC
*JUP*	Autosomal recessive	woolly hair, palmoplantar keratoses. Data suggest that cardiac phenotype is classical ARVC.
		Prevalence of LP/P variants have great geographical differences (Naxos; Greece high, rest low)
*TMEM43*	Autosomal dominant	Almost fully penetrant, with high arrhythmic rate especially in young men. Some left sided involvement.
		Prevalence of LP/P variants have great geographical differences (Newfoundland Canada high, rest low)
*PLN*	Autosomal dominant	Frequent left sided involvement with possible DCM‐like phenotype. Irrespective of phenotype, highly arrhythmic. Classical ARVC risk factors (sex and exercise) do not apply.
		Prevalence of LP/P variants have great geographical differences (the Netherlands high, rest low)
*DES*	Autosomal dominant	Can cause wide spectrum of phenotypic expression. Both cardiac as extracardiac. Should be regarded as highly arrhythmic gene.
		Unknown prevalence but considered rare

Abbreviations: ACM, arrhythmogenic cardiomyopathy; ARVC, arrhythmogenic right ventricular cardiomyopathy; LP/P, likely pathogenic or pathogenic.

It is important to recognize that although inheritance in affected families appears autosomal dominant (with some autosomal recessive forms), ongoing studies suggest oligenic contributions to ACM pathogenesis, with poorly understood genetic factors playing a role in penetrance. The consequence is that penetrance in families is considerably higher than in patients with variants from the general population whose variant is identified as an incidental finding or through population‐based genetic testing. Context of genetic testing is vital to consider in managing these ACM variant carriers. For instance, penetrance in population‐based studies of desmosomal variants is believed to be as low as 1%–6% [15]. The sections below summarize data from family studies.

### 2.1 Desmosomal Genes

#### 2.1.1 PKP2

LP/P *PKP2* variants are most commonly associated with classical right‐dominant ARVC, with individuals with LP/P *PKP2* variants accounting for up to two‐thirds of ARVC cases in cohort studies. Variants are typically loss of function variants and, consistent with haploinsufficiency as a disease mechanism, there is no evidence that specific variants are associated with differences in penetrance or clinical severity. *PKP2*‐associated ARVC is characterized by right precordial T‐wave inversions on ECG, frequent premature ventricular complexes (PVCs), and right ventricular enlargement and dyskinesis. As the majority of ARVC cases are caused by LP/P *PKP2* variants, the general risk factors for ARVC penetrance (i.e. male sex, exercise, multiple LP/P variants) are associated with an increased risk of *PKP2*‐associated ARVC [11, 12]. Likewise, the rate and pattern of progression (i.e., electrical abnormalities on cardiovascular screening before structural involvement) are also similar to that of the overall ARVC population [13]. VAs and PVCs usually originate from the RV with left bundle branch block morphologies [6].

#### 2.1.2 DSP

The first LP/P (autosomal recessive) *DSP* variants were identified in individuals with woolly hair, palmoplantar keratosis and pediatric onset of cardiomyopathy and arrhythmias, known as Carvajal syndrome. Since then, LP/P *DSP* variants have been increasingly identified in ACM families. LP/P *DSP* variants are typically associated with biventricular or left‐dominant cardiomyopathy [16]. Consequently, the term ALVC, biventricular ACM, non‐dilated left ventricular cardiomyopathy and *DSP*‐cardiomyopathy have all been used in association with the disease. For the purpose of this manuscript, we use the term: *DSP*‐cardiomyopathy. *DSP*‐cardiomyopathy is characterized by rare occurrence of right precordial T‐wave inversions, whereas left precordial (most often V4–6) T‐wave inversion may occur, as manifestation of left ventricular disease. Left ventricular involvement is also shown by “ring‐like” late gadolinium enhancement and in the late stage by reduced left ventricular ejection fraction [16]. VAs frequently occur with a right bundle branch block morphology, although left bundle branch block morphologies may occur more often [17]. LP/P *DSP* variant carriers may experience acute episodes of myocardial injury (also called “hot phases”) where patients present with myocarditis‐like symptoms (i.e., chest pain and elevated troponin levels in absence of coronary artery disease) [15]. These episodes are thought to be immune mediated and are associated with an increased risk of VA [16]. A consensus on how to treat these episodes is still lacking (conservative/treating symptoms vs. immunosuppressive medication).

LP/P *DSP* variant carriers have a different risk factor for penetrant disease from classical *PKP2*‐associated ARVC [16]. For instance, in contrast to *PKP2*‐associated ARVC, *female* sex in LP/P *DSP* variant carriers is associated with penetrant *DSP*‐cardiomyopathy and the impact of exercise on penetrance is uncertain [8] (Table 2).

#### 2.1.3 DSG2/DSC2

These 2 genes are considered together, as *DSG2* and *DSC2* encode the two cardiac‐predominant desmosomal cadherins: desmoglein‐2 and desmocollins‐2. LP/P variants in each of these genes contribute to a smaller percentage of ACM. Both *DSG2*‐ and *DSC2*‐cardiomyopathy are phenotypically similar to *PKP2*‐associated ARVC. The limited data available suggest that both *DSG2* and *DSC2* LP/P variants result in precordial T‐wave inversions (most often V1‐3) on ECG, right ventricular dyskinesis due to fibro‐fatty replacement, and may have more biventricular involvement than typically seen in patients with *PKP2* variants [6]. VAs usually originates from the RV with left bundle branch block morphologies. The age of onset in *DSG2*‐associated ACM may be earlier as compared to *PKP2*‐associated ARVC [6] and small reports of *DSC2*‐associated ACM often show an autosomal recessive inheritance pattern [18]. This also holds true for specific *DSG2* variants that can be more prevalent in specific populations [19]. Currently there are not sufficient data available to describe the penetrance of LP/P *DSG2* or *DSC2* variants in detail.

#### 2.1.4 JUP

First described in 1986 as Naxos disease and later definitely determined to be caused by an autosomal recessive/homozygous pathogenic variant in *JUP* [20], these patients, similar to those with Carvajal Syndrome, have woolly hair, palmoplantar keratoses and are at high risk for developing ARVC. The cardiac phenotypic expression is often right‐sided with similar features to *PKP2*‐associated ARVC but a significantly more severe clinical course. Biventricular involvement is not uncommon in these patients. Another important difference between *PKP2* and *JUP* is that homozygous carriers of a pathogenic *JUP* variant show 97% penetrance by adolescence. In summary, *JUP*‐associated ACM (Naxos disease) is an almost completely penetrant disease characterized by high risk of VAs, precordial T‐wave inversions, fibro‐fatty replacement including left‐sided structural abnormalities [20].

### 2.2 Non‐Desmosomal Genes

#### 2.2.1 TMEM43

First described in 2008, *TMEM43* was the first non‐desmosomal gene identified to cause ARVC [2] with one specific variant (p.Ser358Leu) considered definitively pathogenic. This founder variant is highly prevalent in Newfoundland but observed worldwide. *TMEM43*‐cardiomyopathy is characterized by severe arrhythmias especially in young men, poor R wave progression (more common than precordial T‐wave inversions), and dilatation of the left ventricle [21]. The p.Ser358Leu variant is nearly fully penetrant in males and is associated with a very high risk of VA and SCD [21] and exercise is associated with worse clinical outcomes [22]. As such, *TMEM43*‐cardiomyopathy should be considered a highly malignant disease and early consideration of ICD implantation indicated for at‐risk male *TMEM43* variant carriers.

#### 2.2.2 PLN

First described in 2006, the p.Arg14del *PLN* variant is a Dutch founder variant with a mixed arrhythmogenic and heart failure phenotype [3]. In contrast to the overall ARVC population, the general risk factors (i.e., male sex and exercise) do not apply for individuals harboring the p.Arg14del *PLN* variant [23, 24]. *PLN*‐cardiomyopathy is characterized by micro voltages and precordial T‐wave inversions on ECG, high arrhythmic risk, and a relatively high risk of biventricular cardiomyopathy and end‐stage heart failure as compared to other ACM genes.

#### 2.2.3 DES

*DES* is a unique gene in the spectrum of ACM genes, as LP/P variants have been associated with phenotypes across the spectrum of cardiomyopathy morphofunctional subtypes, often associated with proximal and/or distal skeletal myopathy. Studies have reported up to 80% of individuals with LP/P *DES* variants will develop a cardiac phenotype [4]. ACM associated with LP/P *DES* variants itself is clinically heterogeneous as cases with biventricular, left‐dominant, and right‐dominant ACM have been described, although dilated and restrictive cardiomyopathies appear the predominant phenotypes. Notably, certain variants are associated with distinct phenotypes. Patients with LP/P *DES* variants exhibit a high burden of advanced cardiac conduction disease, frequently necessitating cardiac implantable electronic device implantations [4]. While less common, VA and heart failure events, such as hospitalization, left ventricular assist device implantation and transplantation, remain important outcomes, with significant impact on patient survival. Nonetheless, data on natural history of *DES* remain limited, and there are currently no genotype‐specific risk stratification tools to guide clinical decisions.

### 2.3 Potential ACM genes

Although the above‐mentioned genes are moderately or definitely associated with ARVC [14], several other genes are worth noting, which currently have too limited data for full characterization of penetrance but have been linked to ACM. Indeed, cases who harbored LP/P Filamin C (*FLNC*), Sodium channel protein type 5 subunit alpha (*SCN5A*), Lamin A/C (*LMNA*), Cadherin‐2 (*CDH2*), Catenin alpha 3 (*CTNNA3*), Tight junction protein 3 (*TJP1*) have been linked to ACM [25]. Consequently, if an ACM patient is considered “gene‐elusive” (i.e., does not harbor a LP/P variant in genes with moderate or definite evidence of ACM (ARVC) causation [14]), clinicians should consider testing for the above‐mentioned genes.

**Table 2:** Current VA risk calculators available.

Risk calculator	Inclusion criteria	Pros	Cons	Variables needed
Updated 2019 ARVC risk calculator [11]	Definite ARVC diagnosis AND no prior VA	Has been externally validated [9] and has been shown to adequately predict risk of first VA longitudinally [26]	Has been shown to poorly predict risk in LP/P *DSP* carriers [7]	Age, sex, cardiac syncope, number of precordial and inferior T‐wave inversions, PVCs/24h, history of non‐sustained VT, RVEF, PVS (optional)
Life‐threatening ARVC risk calculator [27]	Definite ARVC diagnosis AND no prior life‐threatening VA	Enables risk prediction for life‐threatening VAs	Has not been externally validated. Has not been shown to adequately predict risk longitudinally	Age, sex, number of precordial and inferior T‐wave inversions, PVCs/24h
*PLN* risk calculator [24]	Harboring the *PLN* p.Arg4del variant	Genotype‐specific risk calculator. Can be used in phenotype negative carriers. Has been shown to adequately predict first VA longitudinally [28]	Has not been externally validated.	Age, number of precordial and inferior T‐wave inversions, PVCs/24h, history of non‐sustained VT, LVEF
*DSP* Risk Score [8]	Harboring a LP/P *DSP* variant	Genotype‐specific risk calculator. Can be used in phenotype negative carriers. Has been externally validated	Has not been shown to adequately predict risk longitudinally	Sex, PVCs/24h, history of non‐sustained VT, RVEF, LVEF

Abbreviations: ARVC, arrhythmogenic right ventricular cardiomyopathy; LP/P, likely pathogenic or pathogenic; LVEF, left ventricular ejection fraction; PVS, programmed ventricular stimulation; RVEF, right ventricular ejection fraction; VA, ventricular arrhythmias; VT, ventricular tachycardia.

## 3 (Lack of Gene‐Specific) Diagnosis of ACM

The first diagnostic criteria for ACM were proposed by a task force of experts in 1994, which were subsequently updated in 2010 [29]. It is, however, important to note that these “task force criteria” (TFC) for ACM were only developed for ARVC, not ALVC nor biventricular ACM and did not incorporate specific genotype. Although the 2010 TFC for ARVC are validated [30], this study was performed in mostly LP/P *PKP2* variant carriers. The 2010 TFC do not perform well across ACM genotypes [15]. For instance, a recent publication showed that LP/P *DSP* variant carriers can present with VAs without fulfilling current diagnostic criteria for ARVC [15]. A recent effort has been made to develop criteria based upon expert opinion to encompass all subtypes of ACM [31]. The resulting criteria yielded good diagnostic potential for LP/P *DSP* carriers [32], but unfortunately also had a poor discriminatory potential against ACM phenocopies [33]. Consequently, there is an enormous need to develop evidence‐based gene‐specific approaches to identifying phenotypic onset and managing patients with their specific genotype‐cardiomyopathy *before* VAs occur.

## 4 Management of Individuals At‐Risk for ACM

As ACM is a genetic cardiomyopathy, relatives of ACM patients are at‐risk for developing ACM. Guidelines recommend cascade genetic testing and cardiac screening in family members of ACM patients with an identified LP/P variant, but guidelines are not genotype‐specific [5]. In families where no LP/P variant is identified in the proband, all first‐degree relatives are recommended to undergo longitudinal cardiac evaluation. Although some differences exist between guidelines, there is an agreement that evaluation of relatives should begin by the age of 10–12 years and repeat evaluations be performed at 1–3‐year intervals and include at least an ECG, Holter monitoring, and an imaging modality (either echocardiography or CMR) [5].

Studies have shown that the yield of a baseline cardiac evaluation in at‐risk relatives is approximately 33% in relatives of patients with ARVC, with a marked age‐related penetrance [13]. The incidence of definite ARVC diagnosis in relatives peaks between the 20–30 years of age with pediatric cases less common but certainly observed, particularly at specialized ACM centers, justifying initiation of screening by puberty [34]. The yield of serial evaluation has been shown to be approximately 33% at 4 years of follow‐up [13]. Unsurprisingly, the yield of serial evaluation is highly dependent on presence or absence of abnormalities at first evaluation. Therefore, to allocate resources to those who are at a higher risk of developing ACM and to limit clinical follow‐up in those who have a low risk of developing ACM, a new screening algorithm was proposed that refines follow‐up based on clinical phenotype (i.e., having an additional minor TFC in addition to the major family history criterion), presence of symptoms, and age (20–30 years of age are in need for more closer follow‐up) [13]. It is important to note that the management of at‐risk relatives has been studied in the *overall* ACM population with no focus on underlying genotypes. This introduces yet another challenge, as clinicians frequently face an important question of when and how to screen at‐risk relatives of ACM patients with different underlying genotypes, corresponding to dissimilar risks of VA (and other outcomes) depending on the gene affected [6, 8, 23, 24]. Although no genotype‐specific family screening studies have been published, both the *DSP* [8] as well as the *PLN* p.(Arg14del) [24] risk score can be utilized in gene positive relatives which contrasts with the ARVC risk calculators [11, 27] where definite ARVC diagnosis was the inclusion criterion (Table 2). This enables clinicians to determine the risk of VA in these relatives and highlights differential risk factors but leaves clinicians with the question on when and how to safely screen these relatives longitudinally. Such studies are strongly recommended [8].

## 5 Management of Patients Diagnosed with ACM

To date, there is no curative treatment for ACM. The management strategy in patients with ACM is focused on: (1) risk stratification for implantable cardioverter defibrillator (ICD) placement; (2) medical management; and (3) potentially limiting exercise (i.e., slowing disease progression) [5]. There is increased evidence that genotype influences each.

### 5.1 Risk Stratification for ICD Implantation

Both primary and secondary prevention of SCD are key to ACM management. A growing body of evidence shows that both the absolute risk of VA/SCD and risk factors for these outcomes vary across genotypes. Thus, VA risk stratification and management of ACM patients should center on the underlying genotype and incorporate individualized, genotype‐specific risk factors to guide clinical decisions.

Despite improvements of ICD therapy, including arrhythmia discrimination, ICDs are still associated with a significant risk of complications. Thus, optimal patient selection is necessary to ensure that those in need for an ICD receive one, whilst those who are at a low risk of VA are not recommended an ICD. To enable appropriate patient selection, risk calculators and other approaches to individualized risk prediction for ACM have been developed in recent years and are discussed in detail below.

Decades of research by the ARVC community set the stage for the development of the first ARVC “risk calculator” in 2019 (www.acm-risk.com) [20]. This model is designed to estimate risk of incident VA in patients who fulfill 2010 TFC for ARVC. The VA calculator [11] has been externally validated [9], been shown to perform adequately in athletes [35], and has been optimized for longitudinal follow‐up [26]. Additionally, programmed ventricular stimulation [36] has been added as an optional variable in the ARVC risk calculator. Additionally, a life‐threatening VA calculator has been developed, but has not yet been validated [27] (Table 2).

It is, however, important to note that these models, as well as the previous research done to identify risk factors, included the *overall* ARVC population, with limited stratification by genotype. Efforts to examine the gene‐specific performance of the ARVC risk calculator established that it performs adequately in patients with definite *PKP2*‐associated ARVC [9], overpredicts VA risk in gene‐elusive ARVC patients [9], and performs poorly in patients with ARVC with a LP/P *DSP* variant [7]. Another limitation of the ARVC risk calculator is that it is developed and validated in patients with ARVC according to the 2010 TFC, which limits risk stratification in genotype‐positive, phenotype‐negative family members. Consequently, gene‐ and/or variant specific risk calculators (i.e., *PLN*‐p.Arg14del [24, 28] and *DSP* [8]) have been developed and can also be utilized in appropriate genotypes irrespective of clinical phenotype (www.acm-risk.com). Notably, these genotype‐specific models enable individualized risk prediction in genetic forms of disease, which tend to manifest with VA early in the course of the disease. Nonetheless, all these calculators estimate only the risk of VA and not that of other adverse events (e.g., HF‐associated events), which can be the cardinal manifestation in certain ACM forms (e.g. *DSP* and *PLN*). As such, further efforts are necessary to develop models for prediction of non‐arrhythmic events in genetic forms with substantial risk for the given outcome.

### 5.2 Medical Management

Antiarrhythmic drugs are often prescribed to reduce the risk/burden of VAs [5]. Despite lacking randomized controlled trials for the use of antiarrhythmic drugs in ACM, several studies have tried to retrospectively assess the safety and efficacy of antiarrhythmic drugs in ACM. These studies have conflicting results, which can be explained by the high (genetic) heterogeneity of the study population, and differences in endpoint definitions and medication assessed [37].

Vital translational evidence suggests that specific antiarrhythmics can reduce the risk of VA, likely in a gene‐specific manner. Specifically, in a *PKP2* cardiac conditional knockout mouse model (PKP2cKO), adrenergic stimulation triggered by isoproterenol infusion, or by exercise, induced excess sarcoplasmic reticulum calcium release through RyR2 channels, which promote arrhythmias [38]. Patients with catecholaminergic polymorphic ventricular tachycardia (CPVT) (a genetic disease with bidirectional or polymorphic VTs caused by LP/P variants in *RYR2* or *CASQ2* genes) [39] showed reduction of VA burden with flecainide, which has RyR2 blocking activity in addition to the known sodium channel blocker effect [40]. Remarkably, the use of flecainide eliminated VA occurrence in the PKP2cKO mouse model [38], suggesting it may have similar antiarrhythmic potential in human *PKP2*‐associated ARVC patients. However, data on flecainide in LP/P *PKP2* variant carriers is currently limited, and results from a randomized controlled trial are pending (NTC03685149).

Currently, only beta‐blockers are recommended to reduce the risk of VA in ARVC (preferably a long‐acting, cardio‐selective such as metoprolol) [5]. The evidence behind this approach is, however, limited and without evidence to prevent SCD in ACM. Rather, the rationale for this is that beta‐blockade has been shown to lower the risk of SCD in various patient populations with structural heart disease, prior myocardial injury, and heart failure.

### 5.3 Exercise

As mentioned above, the only modifiable factor associated with disease penetrance is exercise [12]. Consequently, limiting exercise in ARVC patients and at‐risk relatives has historically been recommended in guidelines in an effort to slow disease progression [5]. Indeed, limiting/lowering exercise has not only been shown to decrease the arrhythmic burden in patients with ARVC [41], but has also been shown to be protective for genotype‐positive/phenotype‐negative relatives of ARVC patients in developing penetrant disease as well as VAs [42]. However, these studies have been performed in an *overall* ARVC population which consisted disproportionately of patients with LP/P *PKP2* variants. Recent studies have investigated whether exercise is associated with penetrant disease and/or VAs in individuals harboring pathogenic *PLN* and *TMEM43* variants, and gene‐elusive individuals with ARVC. Exercise in patients with *TMEM43*‐associated ARVC and in gene‐elusive ARVC patients indeed proved to be associated with VAs [22, 43]. In contrast, a recent study found that exercise does not influence the development of penetrant disease, VA, or HF events in individuals with the Dutch founder *PLN*‐p.(Arg14del) variant [23]. Collectively, these findings demonstrate that exercise as a modifiable risk factor for ACM penetrance has a gene‐specific effect and recent guidelines suggest that shared decision‐making for exercise incorporate a gene‐specific approach [44]. From a practical perspective, the major guidelines in ACM management recommend to counsel adolescent and adult individuals who are genotype‐positive/phenotype‐negative that competitive or frequent high‐intensity endurance exercise is associated with an increased risk of developing ARVC and VAs [5, 45]. Consequently, these genotype‐positive/phenotype‐negative individuals are recommended to limit their exercise to mild‐to‐moderate physical activity for up to 150 min per week as that is considered safe [45]. However, these recent findings suggest that exercise restrictions in individuals harboring the *PLN*‐p.(Arg14del) variant may not be advised in mild to moderately active carriers without any known risk factors for VAs, albeit caution is warranted in those with known risk factors for VA [23]. Of note, to err on the side of caution, exercise restriction should be advised in those genotypes in which currently no data is available regarding exercise and adverse events.

### 5.4 The Potential of Gene Therapy

Recently, preclinical data on the effectiveness of gene therapy to arrest the progression of the disease in *PKP2*‐associated ARVC have paved the way for ongoing Phase I clinical trials and to a new potential therapeutic approach to treat ARVC [46, 47]. Gene therapy in *PKP2*‐ associated ARVC is based on adeno‐associated virus (AAV) vectors that act as transporters to deliver the wild‐type *PKP2* gene to the heart. AAVs hold recombinant deoxyribonucleic acid (DNA) and are replication defective, resulting in a nontoxic gene transfer into human cardiomyocytes. In contrast to wild‐type AAVs, the viral genome sequences (encoding for viral replication) are replaced with a functioning human gene (e.g. *PKP2*). As such, currently all AAVs used in gene therapy can be regarded as a DNA‐carrier in which a functioning human gene is transported. Not all ARVC genetic forms are good targets for gene therapy, since only genes with a size < 5.0kb DNA can be successfully inserted in the vector, making *PKP2*‐associated ARVC an ideal candidate. AAV‐mediated delivery of a wild‐type gene is effective for loss of function mutations, as in the case of *PKP2*‐associated ARVC; additionally, delivery of the *PKP2* gene in a normal murine heart did not cause a deleterious effect [46], addressing the concern whether an excess of *PKP2* protein expression could be harmful.

A recent study [46] showed that AAVrh74‐*PKP2* delivery in the PKP2cKO mouse model resulted in 100% survival for > 5 months (compared to 100% mortality after 40–50 days in untreated PKP2cKO animals), prevented RV dilation, arrested LV cardiomyopathy progression and significantly reduced the arrhythmia burden. Importantly for the translational aspects to potential clinical application, gene therapy was effective also in arresting the progression of the mouse phenotype even when delivered after disease onset. These results were supported by a study which used the same mouse model and employed an AAV9‐*PKP2* vector [47]. Additionally, a different genetically modified mouse model harboring a prevalent RNA splice site mutation (IVS10‐1G>C) in humans, and found that AAV9‐*PKP2* gene therapy reduced the pathological deficits when administered at birth or after disease onset [48]. At present, all three therapeutics (Rocket Pharmaceuticals' RP‐A601 [46], LEXEO Therapeutics' LX2020 [48] and Tenaya Therapeutics' TN‐401 [47] used in these studies have US FDA approval for Phase I/II clinical trials for safety and efficacy testing in patients, highlighting the promising progress of AAV‐mediated gene therapy for ACM.

Despite the encouraging progress discussed above, robust gene transfer in human myocardium, remains an important challenge. Failure of CUPID‐2 (gene therapy trial performed in patients with heart failure and a left ventricular ejection fraction < 35%, which had showed no benefit of gene therapy) and associated clinical trials, has largely been attributed to this issue [49]. For ACM gene therapy, achieving a high gene transfer efficiency to ensure a substantial percentage of corrected cardiomyocytes seems crucial. Indeed, a dose‐dependent effect of *PKP2*‐associated ARVC gene therapy was observed with the clinically effective dose anticipated to be in the high 10^13^ or low 10^14^ vg/kg range [46, 47]. This dose is at the threshold of immunogenic risk and hepatotoxicity and higher dosages are therefore currently not considered safe, although this is an evolving field where refinements in the immunosuppression protocol are still being made and evaluated. At the same time, encouraging progress has been made with the development of more potent AAV variants that might help to overcome this issue. For instance, compared to AAV9 and AAVrh74 used in the preclinical studies, a new AAV9 variant, MyoAAV4A, has significantly higher transduction efficiency in heart with lower efficiency in liver, enabling a lower effective dose [50]. Furthermore, local delivery methods such as antegrade intracoronary injections and retrograde coronary vein injections represent important alternatives, as both reportedly achieve global cardiac gene transfer at lower doses compared to systemic intravenous administration [51].

## 6 Conclusions

ACM is a complex and heterogeneous disease characterized by incomplete penetrance, variable phenotype, and gene‐specific differences in both disease expression and outcomes. Gene‐tailored diagnostic criteria and family screening protocols are necessary to enable early diagnosis. The current management strategy of ACM targets (1) slowing disease progression; and (2) preventing SCD. The need for a genotype‐informed approach to risk stratification and management has become increasingly apparent. Gene‐ and founder variant‐specific risk calculators have been developed which can assist physicians with tools for making evidence‐based individualized VA risk assessments. Although currently no curative treatment for ACM is available, flecainide is a promising antiarrhythmic drug and gene therapy has the potential to further fill this gap, particularly for *PKP2*‐associated ARVC, with the first clinical trials now underway.

## Disclosure

G.J.J.B. report ownership interest in PacingCure BV. D.P.J. has received payments as a consultant from Lexeo Therapeutics, Tenaya, Alexion, Alnylam, Attralus, BridgeBio, and Novo Nordisk. All other authors have nothing to disclose.

## Data Availability

Data sharing not applicable to this article as no datasets were generated or analyzed during the current study.
